# Aminated Wood Aerogel via Tannic Acid/Polyethylenimine Co-Deposition for Enhanced Congo Red Removal

**DOI:** 10.3390/gels12090846

**Published:** 2026-09-16

**Authors:** Zhongjian Li, Luohui Wang, Xiaobo Xue, Man Yin, Lin Zhang, Xian Wang, Bing Zhou, Youming Dong, Xiangmeng Chen, Liuting Mo, Cheng Li

**Affiliations:** 1 College of Horticulture; College of Landscape Architecture; College of Forestry, Henan Agricultural University, Zhengzhou 450046, China; 2 College of Materials Science and Technology, Nanjing Forestry University, Nanjing 210037, China; 3 College of Pharmacy, Changsha Medical University, Changsha 410219, China; 4 School of Forestry, Guangxi University, Nanning 530004, China

**Keywords:** wood aerogel, tannic acid, functionalization, adsorption mechanism, Congo red

## Abstract

Wood aerogel has emerged as a highly promising substrate for advanced adsorbents due to its green nature, low cost, high porosity, and unique three-dimensional (3D) interconnected network structure. Harnessing forest resources for developing high-performance aerogel materials is crucial for tackling organic dye pollution. This study presents a novel aminated wood-based aerogel engineered through the co-deposition of tannic acid (TA) and polyethylenimine (PEI) on a cellulose skeleton. The fabrication involved a top–down delignification process to create a porous wood aerogel framework, followed by the in situ loading of TA and the grafting of amino-rich PEI, resulting in the final TAPI-DW composite. Benefiting from the abundant active sites deposited on the aerogel’s hierarchically porous surface and the grafted –NH_2_ groups, TAPI-DW demonstrated an exceptional adsorption capacity for the anionic azo dye Congo red (CR). The adsorption equilibrium was achieved within approximately 6 h, with a lower pH environment promoting removal efficiency. Coexisting ion experiments indicated that the introduction of Ca^2+^ ions dramatically enhanced the CR adsorption capacity from 168.71 mg·g^−1^ to 301.14 mg·g^−1^. This superior capture performance is attributed to the synergistic interplay of the aerogel’s aligned microchannels (derived from the native wood structure) for rapid mass transfer and the intensive chemical interactions, including electrostatic attraction, hydrogen bonding, and π-π stacking between CR molecules and the functional groups (–NH_2_ and –OH) on the 3D skeleton. The Freundlich model fitting suggests a complex multilayer adsorption process on this heterogeneous wood aerogel surface. This work establishes a green and sustainable pathway for fabricating high-value biomass aerogel materials that show promise as candidates for the efficient remediation of dye-contaminated water. Further studies on reusability and long-term stability are needed to fully validate their potential for practical application.

## 1. Introduction

The accelerating pace of industrialization, particularly in sectors like textile dyeing and printing, has led to the discharge of vast quantities of dye-laden effluents into aquatic ecosystems. These synthetic organic dyes are not only aesthetically displeasing, but are also characterized by high structural stability, intrinsic toxicity, and poor biodegradability, posing severe carcinogenic and mutagenic risks to both the environment and public health [1,2,3]. Among them, Congo red (CR) is a representative anionic azo dye that is recalcitrant to degradation and can decompose into even more toxic aromatic amines under specific conditions [4,5,6]. Therefore, the development of cost-effective and highly efficient strategies for removing CR from wastewater is an urgent global challenge. Compared to other methods, adsorption is widely recognized as a superior technique for dye removal owing to its operational simplicity, low energy consumption, and the avoidance of hazardous secondary byproducts [7,8].

Wood, as one of the most important biomass resources, is an ideal candidate for adsorbent substrates due to its abundance, renewability, biodegradability, and ease of modification [9]. Its intricate hierarchical and anisotropic cellular structure provides an ideal template for engineering advanced functional materials. By employing a “top–down” chemical wood nanotechnology, the lignin and partial hemicellulose can be selectively removed from natural wood (NW), leaving behind a 3D cellulose skeleton, which, after a proper drying process like freeze-drying, is transformed into a highly porous material known as wood aerogel (WA) [10,11]. This top–down approach preserves the natural, hierarchically structured cell wall architecture of wood throughout the entire material volume, resulting in a monolithic aerogel rather than a surface-only foam [12]. The freeze-drying step is essential for maintaining this structure, as it prevents the collapse of the delicate cellulosic network [13]. WA preserves the original aligned microchannels of wood but with significantly enhanced specific surface area and exposed functional –OH groups, providing ample sites for functionalization [14,15]. The combination of its complex hierarchical pore structure (from macropores to mesopores) and its inherent surface chemistry makes WA an exceptional scaffold for creating advanced multifunctional materials for environmental remediation [16,17].

Despite these advantages, the adsorption capacity of unmodified WA for anionic dyes remains limited due to the lack of sufficient positive charges on its surface. Tannic acid (TA), a natural, low-cost polyphenol with a high density of phenolic hydroxyl groups, can strongly adhere to the cellulose backbone of the wood aerogel via hydrogen bonding and hydrophobic interactions [18,19]. Although TA itself does not carry positive charges for anionic dye capture, its abundant catechol and pyrogallol groups serve a dual purpose: they not only act as active sites for dye interactions but also function as a secondary reaction platform for grafting amine-rich polymers [20]. Polyethylenimine (PEI), a water-soluble cationic polymer rich in primary, secondary, and tertiary amine groups, is an ideal amination agent [21]. The amine groups of PEI can react with the catechol groups of TA through Schiff base and/or Michael addition reactions, forming a stable, functionalized coating within the wood aerogel channels [22,23]. To ensure robust attachment of this TA-PEI coating to the cellulose skeleton, (3-aminopropyl)triethoxysilane (APTES) was introduced as a molecular bridge. APTES is a well-established silane coupling agent for cellulose functionalization; its hydrolyzed silanol groups condense with cellulose hydroxyls, while its terminal amino groups serve as anchoring sites for subsequent TA deposition via hydrogen bonding and covalent interactions [24,25]. This dual functionality enables APTES to covalently bridge the functional TA-PEI layer to the aerogel scaffold, enhancing the stability and durability of the surface coating. Upon protonation in acidic media, these amine groups (–NH_2_ → –NH_3_^+^) provide the positive charges necessary for electrostatic attraction of anionic dyes like CR. This approach leverages the large specific surface area of the aerogel framework to maximize the deposition of functional layers, creating a high-density, amine-rich interface designed to capture anionic dye molecules such as CR.

In this work, we report for the first time a facile and green strategy to fabricate a novel aminated wood aerogel (TAPI-DW) for the efficient and selective removal of Congo red through the synergistic co-deposition of TA and PEI on a top–down delignified wood aerogel scaffold, with APTES serving as a molecular bridge to enhance interfacial adhesion. Balsa wood was selected as the precursor, delignified, and then freeze-dried to construct the foundational cellulose aerogel framework. Subsequently, a TA/PEI functional layer was co-deposited onto the wood aerogel scaffold via a stepwise in situ process. Compared to previously reported wood aerogel adsorbents, our approach offers several distinctive features: (1) the use of APTES as a molecular bridge to enhance TA deposition on the cellulose surface; (2) the systematic elucidation of the synergistic adsorption mechanism involving electrostatic attraction, hydrogen bonding, and π-π interactions; and (3) the comprehensive investigation of coexisting ion effects, including the remarkable enhancement in CR adsorption by Ca^2+^. The 3D hierarchically porous structure of the WA ensures rapid molecular diffusion, while the highly cross-linked TA-PEI coating provides abundant interaction sites for CR. The effects of pH, temperature, coexisting ions, and humic acid on adsorption performance were systematically investigated for CR as the model anionic dye. For comparative purposes, the adsorption of MB and MO was also evaluated under fixed conditions to demonstrate the material’s charge-selectivity behavior. Furthermore, the intrinsic adsorption mechanism was elucidated through comprehensive characterization and theoretical analysis. This work presents a promising material design strategy for developing sustainable, biomass-based aerogel adsorbents with significant potential for dye removal from aqueous solutions.

## 2. Results and Discussion

### 2.1. Characterization of TAPI-DW

**Figure 1:**
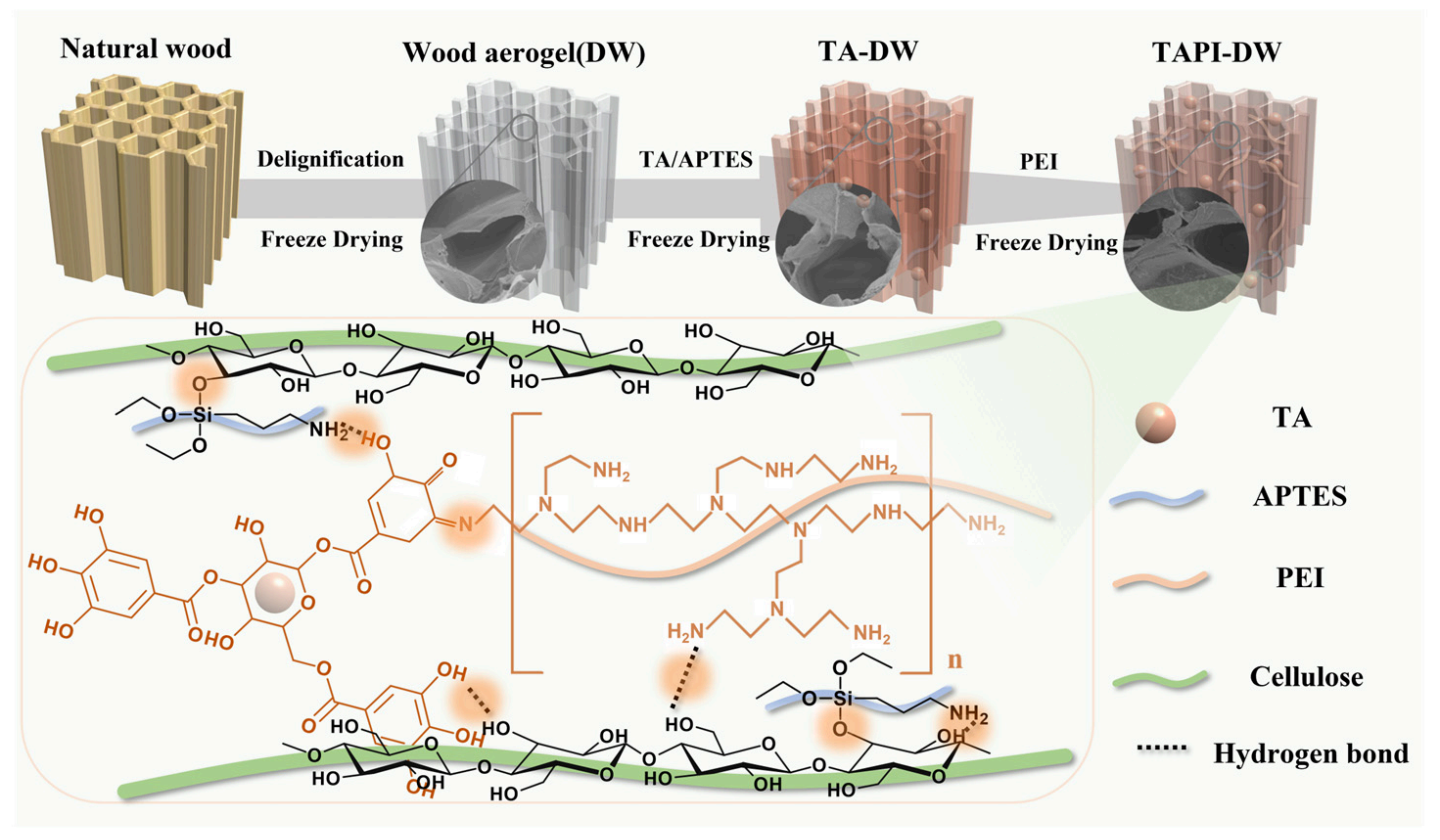
Schematic diagram of the preparation process of TAPI-DW.

To provide a clear understanding of the fabrication process and the underlying chemistry, a schematic illustration of the stepwise synthesis of TAPI-DW is presented in Figure 1. As shown, natural balsa wood undergoes delignification to yield a porous cellulose-based wood aerogel (DW) skeleton. Subsequently, (3-aminopropyl)triethoxysilane (APTES) was employed as a molecular bridge; its hydrolyzed silanol groups condense with the abundant hydroxyl groups on the cellulose backbone, while its terminal amino groups serve as anchoring sites for tannic acid (TA). Finally, the introduction of polyethylenimine (PEI) leads to cross-linking through Schiff-base/Michael addition reactions between the amine groups of PEI and the reactive sites of the TA coating, resulting in the formation of an amine-rich, cross-linked TA-PEI network on the aerogel surface.

**Figure 2:**
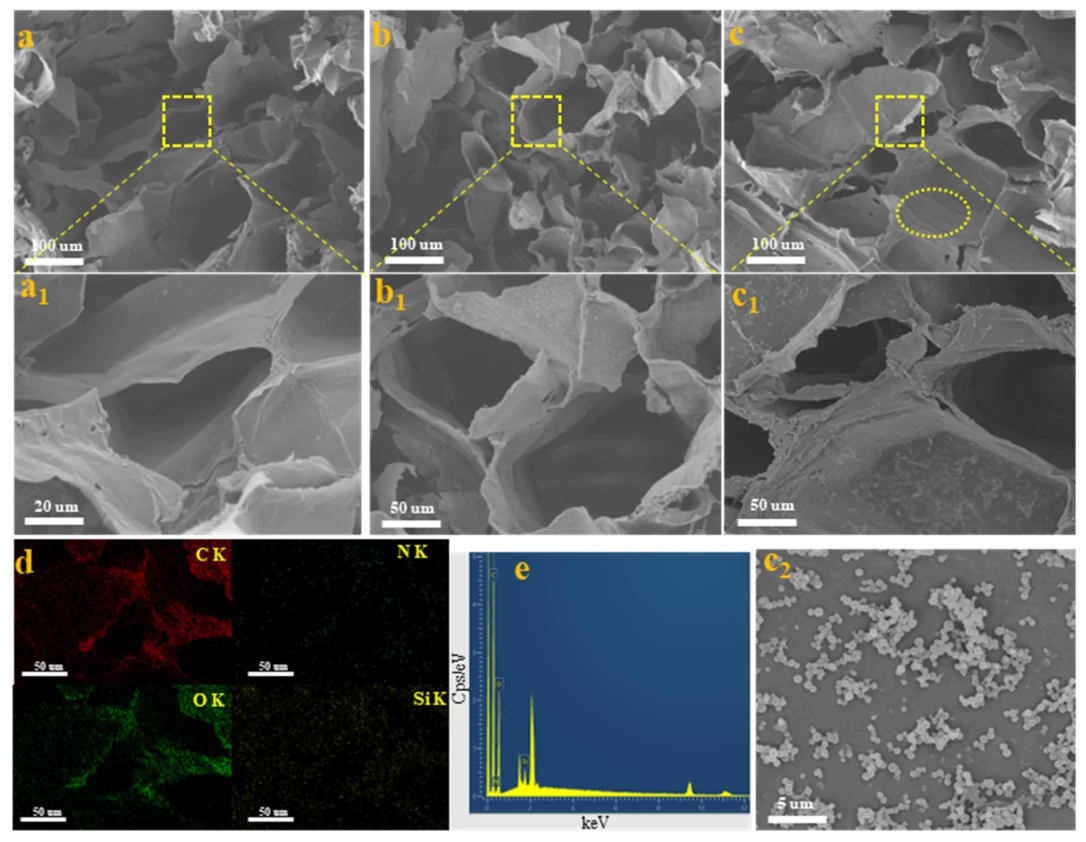
SEM images at different magnifications of (**a**) DW, (**b**) TA-DW, and (**c**) TAPI-DW; (**a_1_**) Magnified SEM image of the area marked by the yellow dashed box in (**a**); (**b_1_**) Magnified SEM image of the area marked by the yellow dashed box in (**b**); (**c_1_**) Magnified SEM image of the area marked by the yellow dashed box in (**c**); (**c_2_**) Magnified SEM image of the area marked by the yellow dashed circle in (**c**); (**d**) EDS elemental mapping (C, N, O, Si) and (**e**) EDS spectrum of TAPI-DW.

Figure 2a–c illustrates the morphological evolution of the wood structure. After the delignification treatment and freeze-drying, the resulting DW, which serves as the foundational cellulose-wood aerogel skeleton, exhibits a relatively dense, smooth cell-wall structure and an open, interconnected macroporous architecture. The SEM images shown in Figure 2a–c were acquired from representative areas of the fractured aerogel samples and are consistent with the typical morphology of freeze-dried cellulose aerogels reported in the literature [26,27]. The visible open channels run through the entire sample block, reflecting the natural hierarchical structure of balsa wood, rather than being localized to a small surface region. After TA modification (TA-DW), the surface roughness increased, exhibiting a particulate texture (Figure 2b). Following the co-deposition of TA and PEI, the surface of the final TAPI-DW composite becomes significantly rougher and is uniformly coated with a dense layer of micro- and nano-particles (Figure 2c). Relevant studies indicate that the incorporation of PEI can induce TA particles deposited on the wood surface to form a typical hierarchical structure (Figure 2c_2_) [28]. These particles are indicative of the cross-linked TA-PEI functional network attached to the aerogel framework. Morphological analysis suggests that TA deposition increased the surface corrugation and roughness, which, combined with the inherent porosity of the WA, enhanced the specific surface area and provided more active sites for pollutant capture. This structural evolution, from a natural template to a functionalized aerogel, is directly conducive to the effective capture of pollutants, thereby improving the dye adsorption capacity of the resulting TAPI-DW.

As shown in Figure 2c,c_1_, the large pore structure of the WA was preserved after the introduction of PEI, although the pore distribution became irregular. This structural feature, characteristic of wood aerogels, contributes to the excellent adsorption efficiency by facilitating the diffusion of dye molecules into the internal pore network. While quantitative pore-structure parameters (e.g., BET surface area and pore-size distribution) were not obtained, the SEM observations clearly reveal that the well-preserved, open microchannel architecture of the wood aerogel facilitates the diffusion of dye molecules into the internal pore network, thereby enabling contact with the functional sites. It is important to emphasize that the primary role of these macrochannels is to serve as mass-transfer highways rather than to provide a high specific surface area for physical adsorption.

EDS analysis (Figure 2d,e) revealed that TAPI-DW was primarily composed of C, N, O, and Si elements, which were uniformly distributed across the TAPI-DW surface and within the pore structures. This uniform distribution indicated a strong interaction between TA and the cellulose skeleton, facilitating its even dispersion on the surface. The uniform distribution of N and Si signals within the pore structure indicates that nitrogen-containing functional groups (derived from APTES and/or PEI) and silane groups (derived from APTES) have been successfully introduced into the cellulose aerogel framework. Importantly, the homogeneous distribution of Si throughout the aerogel matrix, rather than localized aggregation, strongly suggests that APTES is chemically anchored to the cellulose surface via covalent Si–O–C bonds rather than being simply physically adsorbed or subsequently washed away. This observation is consistent with the proposed role of APTES as a molecular bridge that covalently links the functional TA-PEI coating to the wood aerogel scaffold. However, it should be noted that EDS alone cannot distinguish whether the nitrogen signals originate from APTES or PEI, as both contain nitrogen. To better distinguish the nitrogen source, peak fitting was performed on the high-resolution XPS N 1 s spectrum (In Section 2.5); this analysis revealed distinct nitrogen chemical environments, including -N=, -NH-, and -NH_2_ groups. The significant presence of the -NH_2_ component is consistent with the successful grafting of PEI, while the -NH- component likely originates from both APTES and the TA-PEI cross-linking product [29]. This uniform chemical environment ensures a high density of accessible active sites for dye binding.

**Figure 3:**
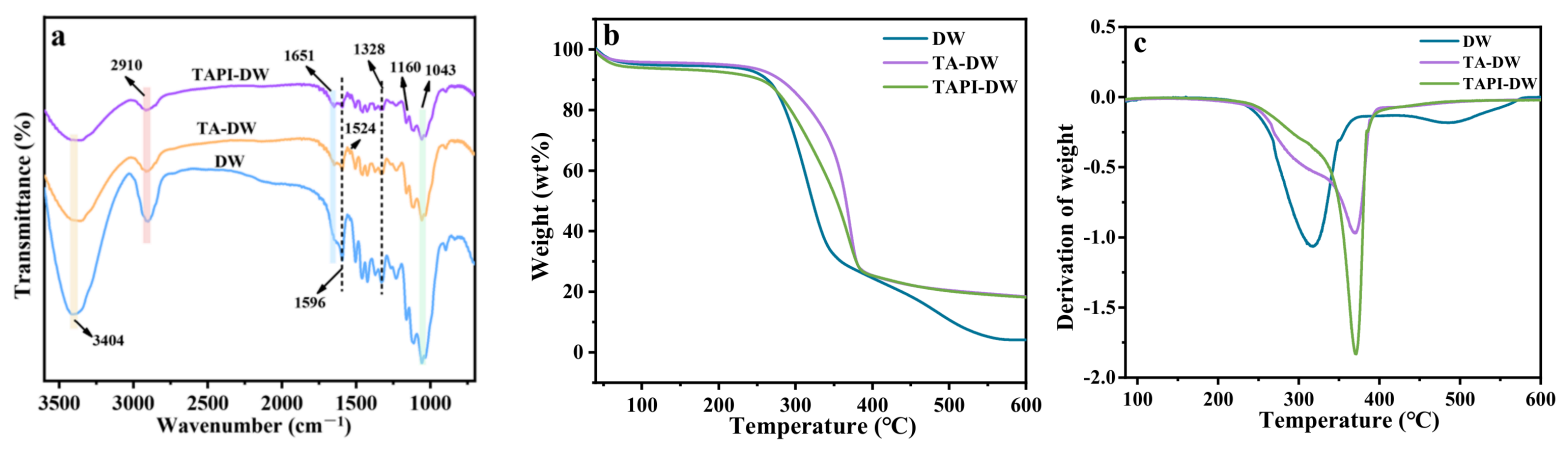
(**a**) FT-IR spectra, and (**b**) TG and (**c**) DTG curves of DW, TA-DW, and TAPI-DW.

As seen in Figure 3a, compared to DW, the peaks in the TA-DW spectrum at 3404, 1596, 1043, and 1328 cm^−1^ exhibited shifts or broadening, which are attributed to O-H, C=C, C-H, and C-C bonds, respectively [30,31]. The peak at 1524 cm^−1^ results from the superposition of vibrations from the inherent functional groups of the WA and the newly introduced aromatic ring of TA, confirming the successful loading of TA onto the cellulose network [32]. The peak at 2910 cm^−1^ is assigned to C-H bonds [33]. Comparative analysis of the spectra after TA and PEI reaction shows a reduction in the C-H peak at 2910 cm^−1^ for TAPI-DW and a shift in the broad -OH peak at 3404 cm^−1^. These changes suggest interactions between the O-H groups of cellulose/hemicellulose and other functional groups introduced by TA/PEI [34]. Most importantly, upon introduction of PEI, the TAPI-DW spectrum exhibits a distinct enhancement at 1651 cm^−1^ (amide I band, C=O stretching) and a new feature at approximately 1556 cm^−1^ (amide II band, N-H bending coupled with C-N stretching), which are characteristic of the formation of amide linkages [35]. These spectral changes provide strong evidence for the occurrence of Schiff base and/or Michael addition reactions between the catechol groups of TA and the amine groups of PEI. Additionally, the peak at 1160 cm^−1^ is attributed to the C-O-C stretching vibration of the cellulose backbone rather than amide formation [36]. The presence of APTES as a molecular bridge is evidenced by the Si 2p peak in the XPS survey spectrum (Figure 3b) and the homogeneous distribution of Si throughout the aerogel matrix (Figure 2d), confirming its role in anchoring the TA-PEI network onto the cellulose skeleton. Together, these spectral features demonstrated the occurrence of a Schiff base reaction between TA-DW and PEI. In summary, the surface-rich oxygen-containing functional groups and -NH_2_ groups on the pore walls of the aminated wood aerogel provided a desirable platform for CR adsorption.

The TG and DTG curves of the materials are presented in Figure 3b,c. The mass loss for all samples below 150 °C is caused by the evaporation of moisture [37]. As shown in Figure 3c, the decomposition rate of all three materials accelerated within the 250–500 °C range, primarily attributed to the rapid degradation of hemicellulose, lignin, and cellulose [38]. The final residual amounts were 4.10%, 18.30%, and 18.14% for DW, TA-DW, and TAPI-DW, respectively. Based on the residual mass differences at 600 °C, the loading amounts of TA and PEI on the wood aerogel were estimated to be approximately 14.2 wt% and 8.5 wt%, respectively. The strong adhesion of TA to the cellulose skeleton is attributed to hydrogen bonding and hydrophobic interactions between the phenolic hydroxyl groups of TA and the hydroxyl groups of cellulose. In contrast, the TA-PEI interaction involves covalent cross-linking via Schiff base and/or Michael addition reactions between the catechol groups of TA and the amine groups of PEI, as supported by the FTIR evidence of amide bond formation (1651 cm^−1^ and 1556 cm^−1^). In the temperature range of 260–380 °C, the TA-treated WA degraded faster than the unmodified WA, while the TAPI-DW, treated further with PEI, exhibited an intermediate degradation rate. From Figure 3c, it can be observed that the maximum thermal degradation temperature increased from approximately 320 °C for the WA precursor (DW) to about 370 °C for both TA-DW and TAPI-DW. This significant increase confirms the successful loading of TA onto the WA skeleton and the effective subsequent modification by PEI.

### 2.2. Adsorption Performance of TAPI-DW

#### 2.2.1. Effect of pH

**Figure 4:**
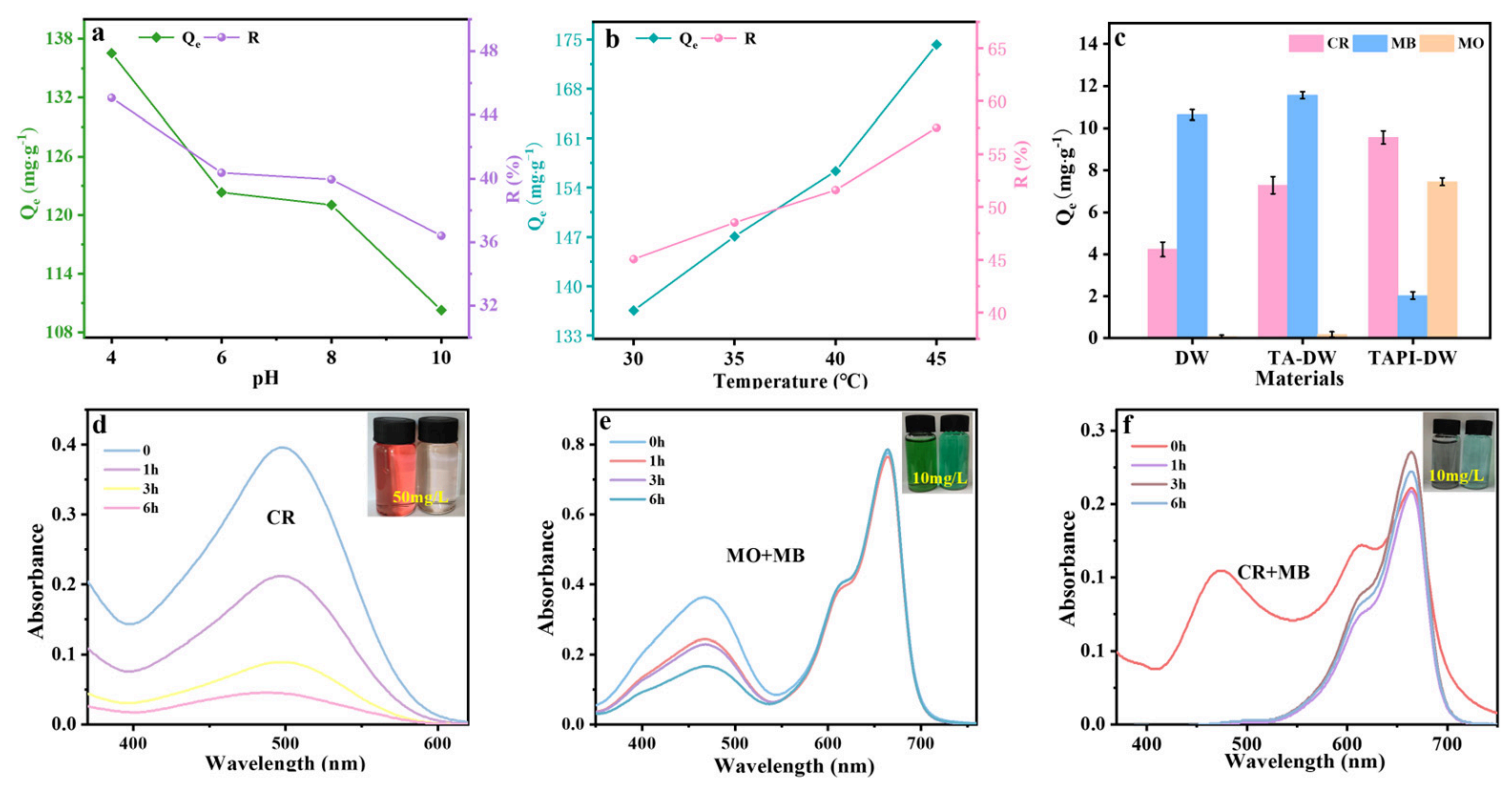
(**a**) The CR adsorption of TAPI-DW effect by pH (C_0_ = 200 mg·L^−1^, adsorbent dosage = 0.033 g, V = 50 mL, t = 6 h, T = 30 °C); (**b**) The CR adsorption of TAPI-DW effect by temperature (C_0_ = 200 mg·L^−1^, adsorbent dosage = 0.033 g, V = 50 mL, t = 6 h, pH = 6.0); (**c**) The adsorption effect of different materials on different dyes (C_0_ = 50 mg·L^−1^ each, adsorbent dosage = 0.033 g, V = 50 mL, t = 12 h, T = 30 °C, pH = 6.0); and (**d**–**f**) The change in absorbance before and after the preferential adsorption of CR and different mixed dyes by the materials (the illustration shows the change in solution color after adsorption for 6 h, C_0_(CR) = 50 mg·L^−1^, C_0_(MB) = 10 mg·L^−1^, C_0_(MO) = 10 mg·L^−1^, adsorbent dosage = 0.033 g, V = 50 mL, T = 30 °C, pH = 6.0).

The acidity and alkalinity of the solution can alter the functional groups on the surface of the adsorbent and affect the state of the dye pollutant molecules. Under acidic conditions, the amino groups in PEI undergo protonation, enhancing the attraction to anionic pollutants [39]. As can be seen from Figure 4a, the adsorption capacity of TAPI-DW for CR decreases with increasing pH. Within the pH range of 4 to 10, the removal rate decreases from 45.1% to 36.4%, and the adsorption capacity drops from a maximum of 136.5 mg·g^−1^ to 110.3 mg·g^−1^. This is likely because, in acidic media, the sulfonic acid group (-SO_3_Na) of the anionic dye molecule ionizes to form -SO_3_^−^, which carries a negative charge [40]. This creates a strong electrostatic attraction with the positively charged protonated amino groups on the WA’s surface, thereby enabling the adsorption of dye molecules [41]. When the pH increases, the negative charge on the Congo red dye decreases, while the concentration of OH^−^ rises. At this point, OH^−^ competes with the dye, leading to a reduction in adsorption capacity. The sharp decrease at pH = 10 may be due to the weakening protonation degree of amino and imino groups at higher pH, resulting in a reduction in active adsorption sites, an increase in electrostatic repulsion, and competition from excessive OH^−^ ions [42]. It can thus be concluded that the solution pH has a significant effect on the CR adsorption performance of the WA-based material, which exhibits higher removal in acidic environments. From a mechanistic perspective, pH-dependent adsorption behavior is primarily governed by changes in the adsorbent’s surface chemistry rather than by CR’s ionization state. The sulfonate groups (-SO_3_^−^) of Congo Red remain strongly ionized across the entire tested pH range of 4 to 10, maintaining its anionic character [43]. Under acidic conditions (pH 4–6), the abundant amine groups (-NH_2_ and -NH-) in the cross-linked PEI network are highly protonated to form -NH_3_^+^ and -NH_2_^+^-, which confer a dense positive charge on the TAPI-DW surface. This generates a strong electrostatic attraction with the negatively charged sulfonate groups of CR, leading to high adsorption [44]. As pH rises from 6 to 10, the degree of protonation of these amine groups gradually decreases, substantially reducing the aerogel’s positive surface charge density. Consequently, the electrostatic driving force for anionic CR capture is significantly weakened. Simultaneously, the increasing concentration of hydroxyl ions (OH^−^) intensifies competition with the anionic dye molecules for the diminishing number of positively charged adsorption sites. Moreover, at higher pH values, increased ionic strength may promote electrostatic screening, further suppressing adsorbent–dye interactions [45,46]. The sharp decrease observed at pH 10 is therefore primarily attributed to the extensive deprotonation of the amine functional groups, which not only reduces electrostatic attraction but also weakens potential hydrogen bonding interactions between the adsorbent and the dye.

#### 2.2.2. Effect of Temperature

The influence of temperature on the material’s equilibrium adsorption capacity is significant. As shown in Figure 4b, as the temperature increased from 30 °C to 40 °C, the adsorption efficiency increased from 45.1% to 57.5%, and the adsorption capacity rose from 136.5 mg·g^−1^ to 174.2 mg·g^−1^. The increase in adsorption efficiency at higher temperatures indicates that this is an endothermic process [47], with maximum adsorption occurring at 45 °C. Higher temperatures favor the endothermic nature of the adsorption process, as increased temperature can alter the solubility and mobility of dye molecules and enhance the activity of the active sites on the surface of the absorbents [48,49].

#### 2.2.3. Preferential Adsorption of Different Dyes

As shown in Figure 4c, the adsorption capacities of the wood aerogel for CR, MB, and MO all increased after TA treatment. Further modification with PEI significantly enhanced the adsorption capacities of TAPI-DW for CR and MO, but reduced its adsorption capacity for MB. The adsorption capacities of TAPI-DW for CR, MB, and MO solutions (each at 50 mg·L^−1^, 50 mL volume) were 9.6 mg·g^−1^, 2.0 mg·g^−1^, and 7.5 mg·g^−1^, respectively, demonstrating that PEI grafting substantially improved the uptake of this anionic dye. The key observation is the opposite effect of PEI grafting on cationic versus anionic dyes: PEI introduces positively charged amine groups that attract anionic dyes (CR, MO) through electrostatic attraction while repelling cationic dyes (MB) [50]. It should be clarified that the MB and MO adsorption experiments were conducted as a preliminary qualitative comparison to assess the charge selectivity of TAPI-DW, rather than as a comprehensive systematic study. Accordingly, detailed investigations of adsorption kinetics, isotherms, and the effects of pH and temperature were conducted exclusively using CR as the representative anionic dye pollutant. CR was selected as the primary model adsorbate because of its environmental significance, molecular complexity, and the richness of its potential interactions (electrostatic, hydrogen bonding, and π-π stacking) with the functionalized aerogel surface. As depicted in Figure 4d, the solution concentration after treatment with TAPI-DW gradually decreased with increasing adsorption time. After 6 h of adsorption, the solution color visibly lightened, and the removal rate reached 88.6%. Regarding preferential adsorption, Figure 4e shows that in the MO + MB mixed solution, the MO concentration decreased progressively as adsorption time increased, and the solution color changed noticeably before and after adsorption. In contrast, the peak intensity of MB in the residual solution showed no significant change. The reason may lie in that the anionic dye MO can interact electrostatically with the positively charged TAPI-DW, while the cationic dye MB experienced electrostatic repulsion, making it difficult to adsorb onto the WA. Furthermore, the smaller molecular size of MO may allow it to penetrate the WA’s porous network more easily, further hindering contact between MB and the surface functional groups. Based on these phenomena, the ionic characteristics of the dye molecules play a more significant role than their molecular size during the adsorption process [51].

As shown in Figure 4f, in the CR + MB mixed solution, the absorbance of CR decreased from 0.146 to 0.001, corresponding to a removal rate of 99.0%, while the absorbance of Methylene Blue decreased only slightly from 0.225 to 0.241. Therefore, the material exhibits preferential adsorption for anions over cations and shows a charge-selective preference for adsorbing CR, which can be attributed to the electrostatic repulsion between the cationic dye and the positively charged adsorbent [52]. Comparing Figure 4e,f, it can be inferred that the observed preferential adsorption arises primarily from electrostatic interactions rather than true molecular selectivity. In the CR + MB mixed system, the change in the MB peak intensity in the residual solution may be because the larger CR dye molecules can quickly interact with the active functional groups on the WA surface through electrostatic interactions, π-π interactions, and hydrogen bonding [53]. After the CR dye is largely adsorbed, MB dye molecules may form hydrogen bonds at specific sites within the WA’s pores [54], leading to the observed change in the residual solution’s peak intensity, which is slightly weaker than that in the MO + MB system. Based on these results, TAPI-DW exhibited good charge-selective adsorption performance for anions and a certain adsorption tendency for cations. Therefore, TAPI-DW holds promise as a multifunctional adsorbent for various dyes, with a preferential affinity for anionic dyes. However, practical application would require further evaluation under real wastewater conditions containing multiple competing components.

### 2.3. Adsorption Kinetics and Isotherms

**Figure 5:**
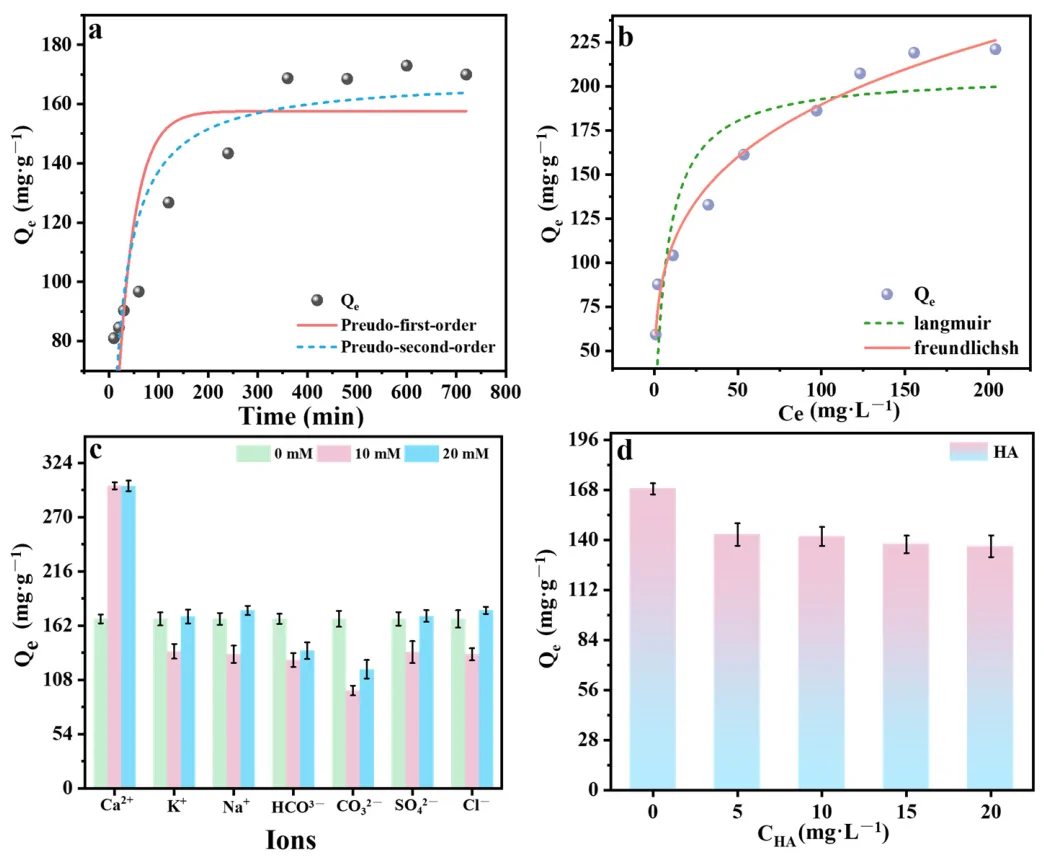
(**a**) Adsorption kinetic model fitting for CR onto TAPI-DW (C_0_ = 200 mg·L^−1^, adsorbent dosage = 0.033 g, V = 50 mL, T = 30 °C, pH = 6.0); (**b**) Adsorption isotherm model fitting for CR onto TAPI-DW at 30 °C (contact time = 6 h, pH = 6.0, C_0_ range = 50–200 mg·L^−1^); (**c**) Effect of different coexisting ions on CR adsorption performance (C_0_(CR) = 200 mg·L^−1^, adsorbent dosage = 0.033 g, V = 50 mL, T = 30 °C, t = 6 h, pH = 6.0, salt concentration = 0–20 mmol·L^−1^); (**d**) Effect of humic acid concentration on CR adsorption performance (C_0_(CR) = 200 mg·L^−1^, adsorbent dosage = 0.033 g, V = 50 mL, T = 30 °C, t = 6 h, pH = 6.0, HA concentration = 0–20 mg·L^−1^).

As shown in Figure 5a, the increase in adsorption capacity of TAPI-DW for CR was slow within 0–30 min under different contact times. However, the rate of increase in equilibrium adsorption capacity was faster before 2 h of adsorption, indicating a rapid adsorption process. This is because, during this period, the abundant macro- and mesopores of the WA provided ready access to a large number of active sites on the pore walls, and the concentration difference between the material surface and the solution was significant, leading to a fast adsorption rate. In contrast, during the 120–360 min period, the adsorption rate slowed down as active sites became largely occupied and the concentration of dye molecules in the solution decreased. After 360 min, the adsorption capacity reached equilibrium. The actual highest adsorption capacity was 173.0 mg·g^−1^, while the theoretically fitted maximum adsorption value was 169.1 mg·g^−1^. The experimental Qe (173.0 mg·g^−1^) was used as the reference value for subsequent experiments (e.g., coexisting ions and HA effects), with a control adsorption capacity of 168.71 mg·g^−1^, showing minor batch-to-batch variation. By further fitting the data with the kinetic equations, relevant kinetic parameters were obtained (Table 1). The theoretically fitted maximum adsorption capacity from the pseudo-second-order kinetic model was closer to the experimental adsorption capacity, and the correlation coefficient R^2^ (0.8172) was relatively higher.

To further elucidate the interaction mechanism between CR molecules and TAPI-DW, the adsorption isotherm was studied using two classical models. The Langmuir model assumes a homogeneous monolayer adsorption process on a surface with equivalent active sites, which implies that once a dye molecule occupies a site, no further adsorption occurs at that site. In contrast, the Freundlich model describes a heterogeneous, multilayer adsorption process on surfaces with non-equivalent active sites, reflecting the energetic diversity of the adsorbent surface. Given that the TAPI-DW surface possesses multiple functional groups (including –OH, –NH_2_, and aromatic rings) and a hierarchical pore structure, the applicability of these two models was systematically evaluated to better understand adsorption behavior.

The adsorption isotherm models were used to fit the relationship between equilibrium concentration (50–200 mg/L) and adsorption capacity at 30 °C. It should be noted that within this concentration range, the adsorption did not reach complete saturation, as the Freundlich model suggests a continuing increase in capacity with concentration. Higher initial concentrations (>200 mg/L) were not tested due to the limited solubility of CR in aqueous solution and the fact that such high concentrations are rarely encountered in real wastewater treatment scenarios. Nevertheless, the Langmuir-fitted q_max_ of 206.83 mg/g provides a reasonable estimate of the material’s maximum capacity under the tested conditions. The fitting data are shown in Table 2. As shown in Figure 5b, the adsorption capacity increases with dye concentration across the entire tested range (50–200 mg/L), and a clear saturation trend is observed at the highest concentration of 200 mg/L. Under low-solution-concentration conditions, the number of active sites on the WA’s extensive pore surfaces is relatively abundant compared to the number of dye molecules. At high concentrations, dye molecules can interact more effectively with these active sites [55]. The constants K_F_ and 1/n in the Freundlich model reflect the adsorption capacity and affinity of the adsorbent. The fitted 1/n value is less than 1, indicating that the adsorption process is favorable [56]. The Freundlich model provides a better fit to the experimental data (R^2^ = 0.9797) than the Langmuir model (R^2^ = 0.7537). The better fit of the quasi-second-order model indicates that the adsorption process is primarily limited by the availability of surface active sites, consistent with strong chemical interactions such as electrostatic attraction, hydrogen bonding, and π-π stacking. While this higher R^2^ value is consistent with a heterogeneous, multilayer adsorption process, it should be noted that model fitting alone does not conclusively prove a specific adsorption mechanism. Nevertheless, the complex surface chemistry of TAPI-DW—featuring multiple functional groups (-OH, -COO^−^, -NH_2_) with different binding energies [57]—and its hierarchical pore structure are both conducive to multilayer adsorption behavior. The comprehensive spectroscopic analysis (FTIR and XPS) results presented below reveal specific interactions, such as electrostatic attraction and hydrogen bonding, which provide the primary evidence for the nature of the chemical reactions during adsorption.

From the perspective of the material’s structural contribution, the well-preserved microchannels derived from the natural wood provide unobstructed pathways for CR molecules to access the densely functionalized pore walls. This mass-transfer effect, rather than physical adsorption within micropores, is the primary structural factor enhancing the overall adsorption kinetics. The significant improvement in adsorption capacity observed after TA/PEI modification, as compared to DW, further confirms that the introduction of abundant -NH_2_ and -OH functional groups plays a decisive role in the chemisorption process. Therefore, the adsorption performance of TAPI-DW is governed predominantly by surface chemical interactions, while the porous architecture serves as a supporting scaffold for efficient molecular delivery. This mechanistic understanding is consistent with previously reported wood-based adsorbents, in which chemical modification, rather than physical texture, dominates the enhancement in pollutant capture. The comprehensive spectroscopic analysis (FTIR and XPS) results presented below reveal specific interactions such as electrostatic attraction and hydrogen bonding, which are the main evidence supporting the nature of chemical reactions in the adsorption process.

### 2.4. Effect of Coexisting Ions and Humic Acid

Furthermore, ionic strength has a significant influence on adsorption performance. CaCl_2_, KCl, NaCl, NaHCO_3_, Na_2_CO_3_, and Na_2_SO_4_ are common soluble salts found in wastewater containing organic dyes. Therefore, it is necessary to evaluate the effect of inorganic salt ions on CR adsorption by TAPI-DW. This study prepared solutions with different concentrations of salt ions (0–20 mmol·L^−1^) to investigate the effect of ionic strength on the adsorption performance. Generally, salt ions can affect adsorbent performance by diffusing onto the adsorbent surface, occupying active sites, or by altering hydrophobic interactions to reduce the solubility of the adsorbate [58,59].

As shown in Figure 5c, the addition of Ca^2+^ increased the adsorption capacity from 168.71 mg·g^−1^ to 301.14 mg·g^−1^ (experimental values), which might be due to Ca^2+^ undergoing electrostatic interactions with dye molecules, or the presence of Ca^2+^ promotes the adsorption of CR onto TAPI-DW through a salting-out effect [60]. Moreover, the addition of CO_3_^2−^ and HCO_3_^−^ inhibited adsorption, reducing the adsorption capacity from 168.71 mg·g^−1^ to 127.82 mg·g^−1^ and 97.44 mg·g^−1^, respectively. The inhibitory effect of CO_3_^2−^ was more pronounced than that of HCO_3_^−^, because HCO_3_^−^ and CO_3_^2−^ compete with CR molecules for adsorption [61], and both can hydrolyze to produce -OH, thereby altering the pH. Among them, CO_3_^2−^ has a greater degree of hydrolysis, producing more -OH and making the solution more alkaline, which reduces the adsorption capacity. This conclusion is consistent with the previously mentioned effect of pH on adsorption performance. The other added ions are neutral and have low hydrolysis degrees, thus their ionic strength has little effect on CR removal, of which the slight inhibitory effect mainly stemmed from weak electrostatic repulsion between the WA material and the dye molecules [62].

To explore the effect of HA on the dye adsorption of TAPI-DW, different concentrations of HA were added to a 200 mg·L^−1^ CR solution. As shown in Figure 5d, when the added amount was 5 mg·L^−1^, the adsorption capacity decreased significantly from 168.71 mg·g^−1^ to 143.14 mg·g^−1^, which can be attributed to competitive adsorption between HA and CR. However, upon further increasing the concentration of humic acid, the adsorption capacity of CR by TAPI-DW remained essentially stable. This is because HA contains functional groups such as carboxyl, which can adsorb CR. However, these functional groups is unfavorable for CR adsorption.

### 2.5. Analysis of CR Adsorption Mechanism by TAPI-DW

**Figure 6:**
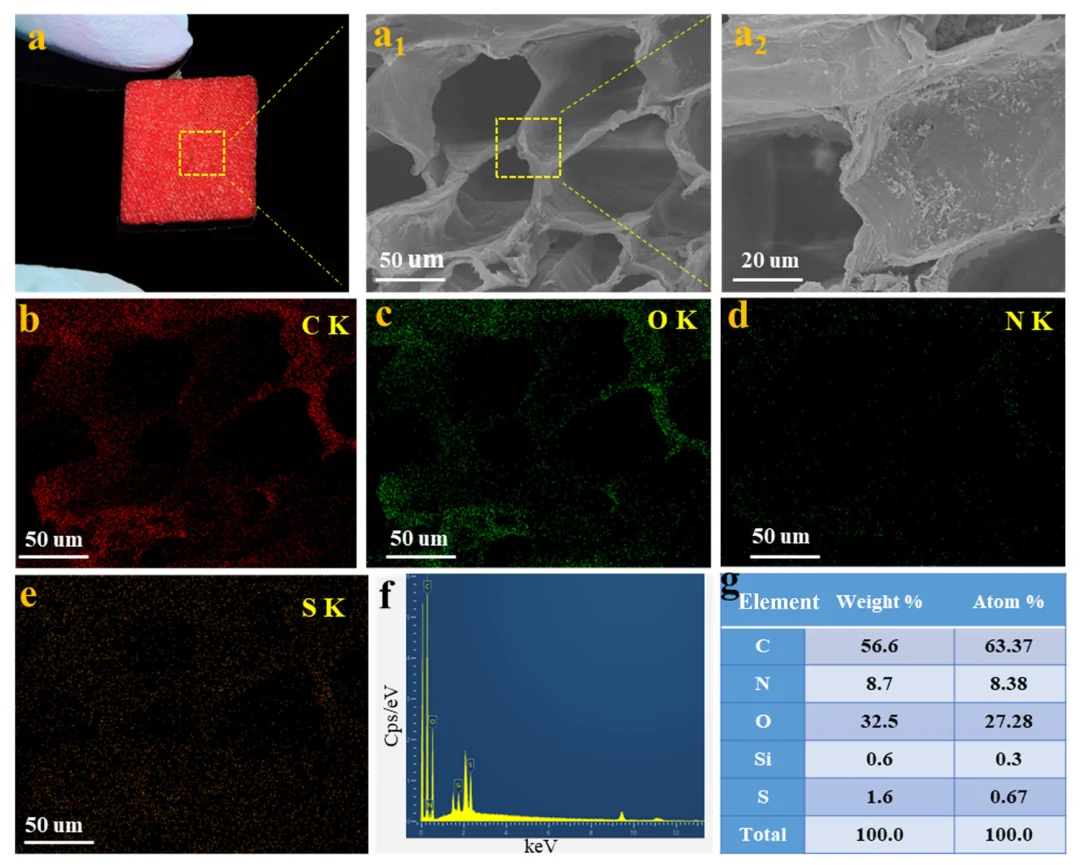
The morphological structure and EDS map of TAPI-DW adsorption CR. (**a**) Macroscopic photos of samples after adsorption CR, (**a_1_**,**a_2_**) enlarged SEM images, and (**b**–**g**) element distribution map of the adsorbed material.

During the adsorption experiments, the TAPI-DW monoliths maintained their structural integrity without visible disintegration, and the reproducible adsorption performance across repeated measurements indicated good material stability under the tested conditions (30–45 °C, pH 4–10, 165 rpm). As shown in Figure 6a, the basic elemental composition of TAPI-DW after dye adsorption was investigated through scanning electron microscopy and EDX analysis of its surface. From Figure 6a–g, the dye molecules were uniformly adsorbed on the WA surface, turning the wood block red, while the hierarchical pore structure remained intact and the loaded particles were clearly visible (a_2_), with the surface becoming rougher. The preservation of the porous network after adsorption highlights the structural integrity of the wood aerogel. The S element was evenly distributed across the WA surface because CR, which contains S, was extensively adsorbed onto the TAPI-DW material [63].

Beyond the structural integrity of the porous network, the macroscopic stability of TAPI-DW during the adsorption experiments deserves comment. Throughout the entire adsorption process (up to 6–12 h under continuous shaking at 165 rpm and 30 °C), the TAPI-DW monoliths retained their original block shape without observable disintegration, macroscopic cracking, or excessive swelling. The kinetic profiles (Figure 5a) exhibited smooth and consistent trends without abrupt fluctuations that would otherwise indicate structural collapse or significant delamination of the functional coating. These observations suggest that the TA-PEI network is sufficiently robust to withstand the mechanical agitation and aqueous environment during a single adsorption cycle. This stability is likely attributable to covalent cross-linking between TA and PEI via Schiff base and Michael addition reactions (as evidenced by FTIR amide bands), which firmly anchors the functional coating to the cellulose skeleton. Nevertheless, it should be acknowledged that these are qualitative indications; systematic evaluation of long-term leaching behavior and coating durability over multiple cycles remains an important direction for future work.

**Figure 7:**
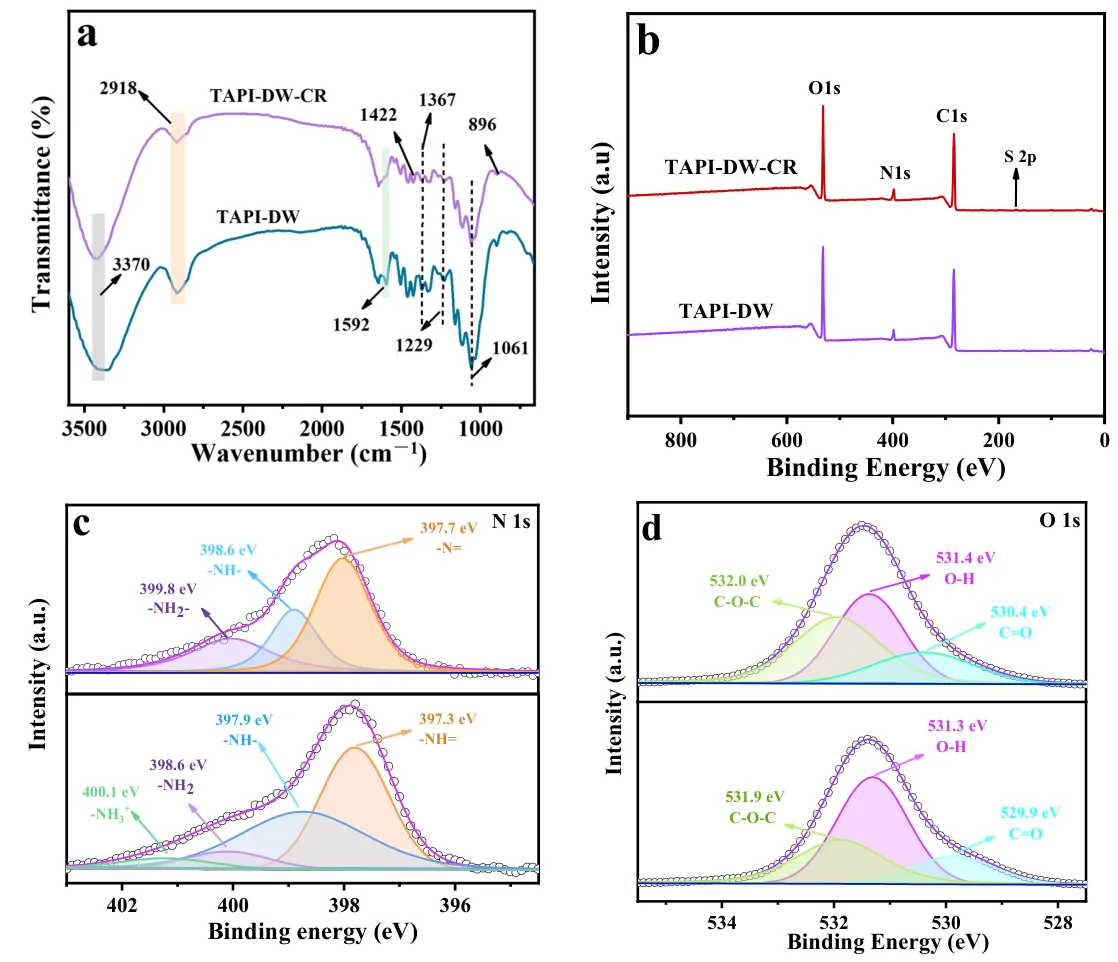
(**a**) Infrared spectrum after TAPI-DW adsorption, (**b**) XPS whole spectrum, (**c**,**d**) N 1 s and O 1 s fine spectrum.

In Figure 7a, the peak at 3370 cm^−1^ indicates the O-H stretching vibration [64]. The changes in the intensity and peak position of the O-H group band before and after adsorption are consistent with hydrogen-bonding interactions between the dye molecules and TAPI-DW. Notably, after CR adsorption, this O-H band shifts from 3370 cm^−1^ to approximately 3385 cm^−1^ with a slight decrease in intensity. This shift clearly indicates hydrogen-bonding interactions between the hydroxyl groups of TAPI-DW and the sulfonate/amino groups of CR molecules. The peak at 2918 cm^−1^ is attributed to the C-H single bonds. The absorption band appearing at 1235 cm^−1^ and the peak at 896 cm^−1^ belong to C-C and C-H bonds, respectively [65]. After CR adsorption, the intensity of the TAPI-DW peak at 1592 cm^−1^ (attributed to N-H bending vibration of amine groups) decreased markedly, indicating the involvement of surface -NH_2_ groups in CR adsorption through electrostatic interactions and hydrogen bonding. This confirms that the active sites grafted onto the WA’s pore walls are crucial for chemisorption. After CR adsorption by TAPI-DW, the peak at 1422 cm^−1^ is attributed to the S=O stretching vibration [66]. New absorption peaks appeared at 1061 and 1367 cm^−1^, which were attributed to –SO and –SO_3_ bonds, respectively. Furthermore, the peak at 1229 cm^−1^ confirmed the presence of –SO_3_ groups.

As shown in Figure 7b, all wood samples possess C 1 s, O 1 s, and N 1 s peaks. After adsorption, the increases in S and N atoms, characteristic of CR, indicate that many dye molecules adsorb on the TAPI-DW surface. As shown in Figure 7c, the XPS spectrum of N 1 s provides further evidence for the CR adsorption mechanism. Before adsorption, the peaks in the N 1 s spectrum of TAPI-DW at 397.7, 398.6, and 399.8 eV are mainly attributed to -N=, -NH-, and -NH_2_-, respectively [67]. However, comparing the N 1 s spectra of TAPI-DW before and after adsorption reveals a new peak at 400.1 eV, which is consistent with partial protonation of amino groups (-NH_3_^+^) [68]. This further supports the occurrence of electrostatic attraction during the adsorption of CR molecules by TAPI-DW. From Figure 7d, the C=O peak shifts from 530.4 eV to 529.9 eV, which may be attributed to interactions between the C=O groups on the TAPI-DW material surface and the functional groups on CR molecules. After CR adsorption by TAPI-DW, changes in the -OH, -NH, and -NH_2_ peaks [69]. Changes in the N 1 s and O 1 s spectra indicate that the amino and hydroxyl groups are the primary interaction sites. Although these spectral changes suggest a strong interaction between the adsorbent and CR, they do not conclusively prove the formation of new covalent bonds (such as Schiff bases) between the adsorbent and CR. Therefore, this mechanism is described as the combined result of strong non-covalent interactions (electrostatic attraction, hydrogen bonding, and π–π interactions) and possible partial covalent bonding.

**Figure 8:**
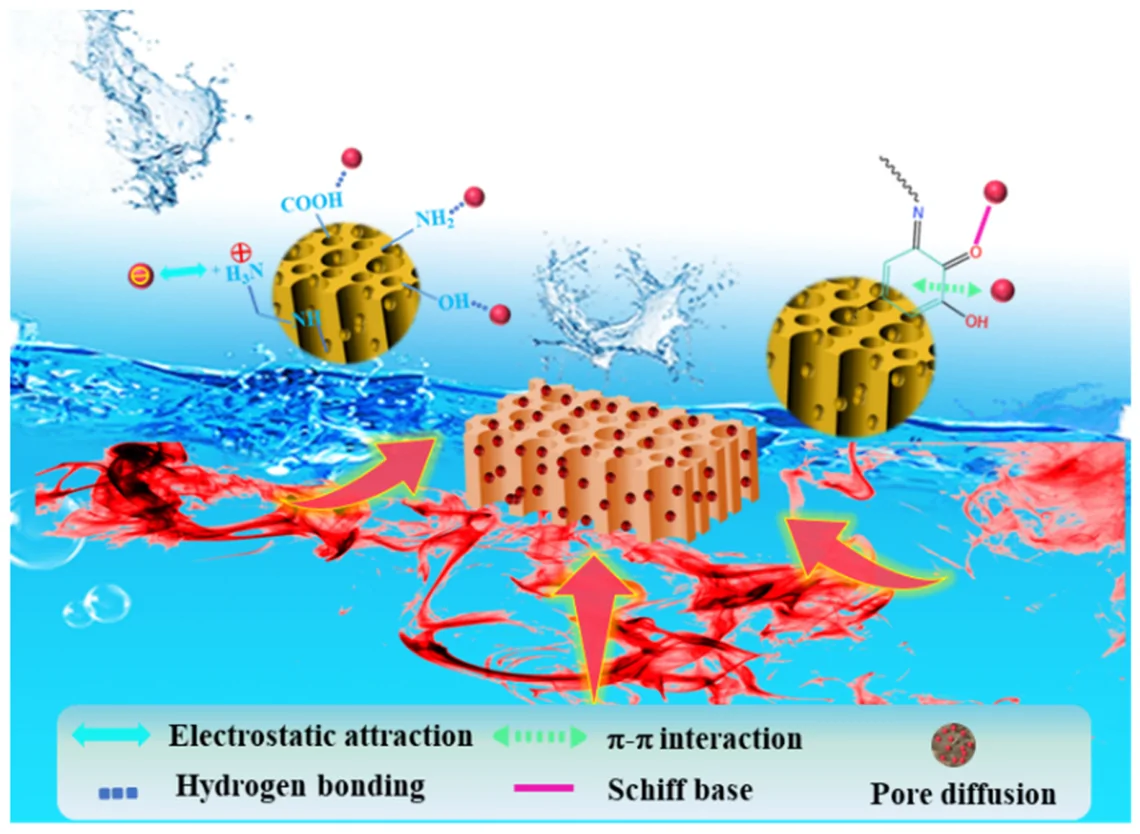
Schematic diagram of the adsorption mechanism of CR by TAPI-DW.

A schematic diagram of the adsorption mechanism is shown in Figure 8. It can be seen that the adsorption mechanism of CR onto TAPI-DW is a synergistic process, combining physical and chemical adsorption. The wood aerogel’s three-dimensional, hierarchical porous structure (macropores, mesopores) provides excellent channels for rapid mass transfer and offers a large surface area for dye molecule capture, while the microporous structure contributes to capillary effects and physical entrapment. Concurrently, the chemical interactions are dominated by electrostatic attraction and hydrogen bonding. The electrostatic interaction between the S=O groups in the anionic dye and the -NH_2_ groups in TAPI-DW is the primary mechanism of adsorption. It is worth noting that the –NH_2_ groups participating in this electrostatic attraction originate from both the PEI coating and the APTES molecular bridge, as APTES also carries a terminal primary amine. Thus, the introduction of APTES not only enhances the interfacial adhesion of the TA-PEI coating but also provides additional amine-binding sites for CR capture. Meanwhile, the hydrogen bonds formed between the -OH and -NH_2_ groups present in the material and the dye molecules also play a significant role. Furthermore, π-π interactions between the aromatic rings of CR and the aromatic structure of TA further contribute to the overall adsorption. This complex multilayer chemisorption, facilitated by the WA’s accessible pore network and its surface functional groups, is well-described by the Freundlich isotherm and pseudo-second-order kinetic models.

### 2.6. Comparison with Reported Congo Red Adsorbents

To contextualize the adsorption performance of TAPI-DW, a comparison with recently reported Congo Red adsorbents is presented in Table 3. The maximum adsorption capacity of TAPI-DW (206.83 mg·g^−1^ from Langmuir fitting, and up to 301.14 mg·g^−1^ in the presence of Ca^2+^) is competitive with many biomass-derived and amine-functionalized adsorbents. While some advanced materials, such as MOF/cellulose aerogel composites, exhibit higher capacities (e.g., 759.21 mg·g^−1^), TAPI-DW offers distinct advantages, including low cost, environmental friendliness, facile preparation from renewable wood resources, and a monolithic form factor that facilitates easy handling and recovery. The adsorption capacity of TAPI-DW is significantly higher than that of unmodified wood aerogel, and many reported cellulose-based adsorbents, demonstrating the effectiveness of the TA/PEI co-deposition strategy.

## 3. Conclusions

In summary, a novel amino-functionalized wood aerogel (WA) adsorbent, denoted as TAPI-DW, was successfully fabricated via delignification, co-deposition of TA and PEI, and a subsequent freeze-drying process. The incorporation of TA increased the surface roughness and introduced abundant functional groups onto the WA’s highly porous cellulose skeleton, thereby significantly enhancing its pollutant adsorption capabilities. Various environmental factors were found to influence its adsorption capacity. Notably, the addition of Ca^2+^ significantly promoted the adsorption of CR, increasing the adsorption capacity up to 301.14 mg·g^−1^. TAPI-DW also exhibited preferential adsorption in various binary mixed-dye systems. The adsorption mechanism is attributed to a synergistic effect: the open microchannel structure of the WA facilitates rapid mass transfer and ensures the accessibility of internal functional sites, while the primary driving forces for dye capture are electrostatic attraction, hydrogen bonding, and π-π interactions between the CR molecules and the -NH_2_/hydroxyl groups on the pore walls. These chemical interactions, together with the physical confinement effect of the porous network, collectively govern the overall adsorption performance. In summary, the TAPI-DW adsorbent exhibits advantages such as low cost, excellent adsorption performance, environmental friendliness, and remarkable selectivity in mixed dye systems, offering a promising new pathway for developing materials to address water pollution.

However, the practical applicability of the TAPI-DW adsorbent requires further investigation. The present study has not systematically evaluated the regeneration performance, adsorption–desorption cycling stability, or the long-term leaching behavior of the TA-PEI coating in aqueous environments. These aspects are critical for assessing the material’s economic viability and operational lifetime in real-world water treatment scenarios. Nevertheless, the covalent cross-linking between TA and PEI via Schiff-base/Michael addition reactions, as evidenced by FTIR analysis (amide I and II bands at 1651 and 1556 cm^−1^), suggests a robust immobilization of the functional coating onto the aerogel skeleton, which offers a promising basis for future cycle-performance studies. We envision that subsequent work will focus on systematic reusability assessments, optimization of regeneration conditions, and evaluation of the adsorbent’s performance under continuous-flow operation to bridge the gap toward practical implementation.

## 4. Materials and Methods

### 4.1. Materials

Balsa wood (density 0.14–0.16 g cm^−3^, Zhuhai Dechi Trade Co., Ltd., Zhuhai, China) size was 20 × 20 × 4 mm^3^. (3-Aminopropyl) triethoxysilane (APTES, 99%), tannic acid (TA, ACS reagent, ≥98%), polyethylenimine (PEI, branched, Mw ≈ 600 Da, 99%), sodium bicarbonate, sodium chlorite, and acetic acid were supplied by Shanghai Macklin Biochemical Technology Co., Ltd. (Shanghai, China). Sodium hydroxide, anhydrous sodium sulfate, sodium chloride, potassium chloride, anhydrous sodium carbonate, anhydrous calcium chloride, and hydrochloric acid were provided by Sinopharm Chemical Reagent Co., Ltd. (Shanghai, China). Methylene blue (MB, C.I. 52015, purity ≥ 95%), Congo Red (CR, C.I. 22120, purity ≥ 85%), methyl orange (MO, C.I. 13025, purity ≥ 90%), and humic acid (HA, technical grade) were purchased from Shanghai Titan Technology Co., Ltd. (Shanghai, China), and Tianjin Guangfu Technology Development Co., Ltd. (Tianjin, China), respectively.

### 4.2. Preparation of Aminated Wood Aerogel (TAPI-DW)

Wood blocks were immersed in an aqueous solution of 3 wt% sodium chlorite (pH adjusted to ~4.6 with acetic acid) and treated at 85 °C for 6 h to selectively remove lignin from the cell walls, leaving the cellulose skeleton intact. The resulting delignified wood blocks were washed three times with ethanol and deionized water to remove all residual chemicals. Subsequently, the wet blocks were frozen at −20 °C for 12 h and then freeze-dried for 24 h. This critical step of ice-templating and freeze-drying preserves the original 3D architecture while endowing the material with the low-density, high-porosity characteristics of a wood aerogel (DW), setting the stage for further chemical functionalization. In this process, the water confined within the delignified wood cell walls forms ice crystals that sublimate under vacuum, leaving behind a three-dimensional interconnected porous network. This freeze-drying route is a standard method for preparing aerogels from hydrogels, as it effectively prevents the collapse of the cellulosic skeleton due to capillary forces during conventional evaporation. The resulting DW exhibits the defining features of an aerogel: high porosity, low density, and a continuous meso- and macroporous network derived from the natural wood template.

For the functionalization of the aerogel, TA (1.0 g) was first dissolved in 400 mL of a hydrochloric acid buffer (pH ≈ 5). Separately, APTES (1.0 g) was added as a potential coupling agent, pre-dissolved in 100 mL of ethanol, and then mixed with the TA solution. The silane groups of APTES are expected to form Si-O-C bonds with the hydroxyl groups on the cellulose skeleton through hydrolysis and condensation reactions, while its amine end groups may interact with TA via hydrogen bonding or covalent linkages. An appropriate amount of the DW was added to this mixture and stirred at 65 °C for 4 h, leading to the in situ deposition of TA particles into the interconnected aerogel network, yielding TA-DW.

After washing and 24 h freeze-drying, the TA-DW was subsequently immersed in a homogeneous PEI solution (1.0 g in 200 mL DI water) and stirred for 6 h to allow for the cross-linking and grafting of the cationic polymer onto the TA-coated skeleton. Finally, the aminated wood aerogel (TAPI-DW) was obtained after repeated washing with hot DI water, −20 °C freezing, and 24 h freeze-drying. For adsorption tests, the monolithic aerogel blocks were cut into four pieces of approximately equal mass.

### 4.3. Material Characterization

The pore structure and surface morphology of the materials were characterized using scanning electron microscopy (SEM, Hitachi Regulus 8100, Hitachi, Tokyo, Japan). Elemental distribution, chemical states of elements and Surface functional groups was characterized by Energy-dispersive X-ray spectroscopy (EDS, Oxford Instruments X-MaxN 50, Oxford Instruments, Oxford, UK), X-ray photoelectron spectroscopy (XPS, Thermo Scientific ESCALAB 250Xi, Thermo Fisher Scientific, Waltham, MA, USA) and Fourier transform infrared spectroscopy (FTIR, Thermo Scientific Nicolet iS50, Thermo Fisher Scientific, Waltham, MA, USA), respectively. Thermal stability was detected by thermogravimetric analysis (TGA, PerkinElmer STA 6000, PerkinElmer, Waltham, MA, USA). Solution pH was adjusted using a pH meter (PHS-3C, Shanghai INESA Scientific Instrument Co., Ltd. (Leici), Shanghai, China). UV–visible spectrophotometer (Shimadzu UV-1900S, Shimadzu, Kyoto, Japan) to evaluate adsorption performance.

### 4.4. Dye Adsorption Experiments and Adsorption Kinetics and Isotherm Models

Methylene blue (MB), Congo Red (CR), and methyl orange (MO) were selected as model dye pollutants to evaluate the adsorption capacity of TAPI-DW. At the same time, DW (unmodified delignified wood aerogel) was used as the reference material. All adsorption experiments were conducted in a thermostatic shaker (MaxQ 4000, Thermo Fisher Scientific, Waltham, MA, USA) at 165 rpm. After reaching adsorption equilibrium, the residual solution was extracted with a syringe and filtered through a 0.24 μm aqueous filter. The filtrate concentration was measured at wavelengths of 497 nm (CR), 664 nm (MB), and 463 nm (MO). All tests were performed in triplicate. The adsorption capacity, adsorption amount at time t, and removal efficiency were used to quantitatively analyze the adsorption performance of TAPI-DW. The adsorption kinetics were modeled using the pseudo-first-order and pseudo-second-order equations, it was followed our previous report [77].

For the adsorption kinetics experiments, TAPI-DW (0.033 g) was added to 50 mL of CR solution with an initial concentration of 200 mg·L^−1^. The mixture was shaken at 30 °C and 165 rpm. At predetermined time intervals (0, 5, 10, 20, 30, 60, 120, 180, 240, 300, 360, 420, and 480 min), 2 mL aliquots of the supernatant were withdrawn using a syringe and filtered through a 0.24 μm aqueous filter. To maintain a constant solution volume, an equal volume of fresh CR solution at the same concentration was replenished after each sampling. The pH of the solution was monitored throughout the experiment and maintained at 6.0 ± 0.1 using 0.1 mol·L^−1^ HCl or NaOH. The residual CR concentration in the filtrate was determined using a UV–visible spectrophotometer (Shimadzu UV-1900S, Shimadzu, Kyoto, Japan) at the maximum absorption wavelength of 497 nm. The calibration curve was established using standard CR solutions in the concentration range of 1–100 mg·L^−1^ (R^2^ > 0.999). Samples with concentrations exceeding the linear range were appropriately diluted before measurement. All experiments were performed in triplicate, and the results are presented as mean ± standard deviation. The adsorption capacity at time t (q_t_, mg·g^−1^) and the equilibrium adsorption capacity (q_e_, mg·g^−1^) were calculated using the following equations:

(1) $$q_{e}\;=\;\frac{\left(C_{0}\;-\;C_{e}\right)V}{m}$$

(2) $$q_{t}=\frac{\left(C_{0}-C_{t}\right)V}{m}$$

where C_0_ (mg·L^−1^) is the initial dye concentration, C_t_ (mg·L^−1^) is the dye concentration at time t, C_e_ (mg·L^−1^) is the equilibrium dye concentration, V (L) is the solution volume, and m (g) is the mass of the adsorbent.

(1) Adsorption of Different Dyes by Different Materials

Solutions of dyes (MB, CR, and MO) were treated with DW, TA-DW, and TAPI-DW at 30 °C for 12 h. These experiments were conducted as preliminary screening tests to evaluate the charge selectivity of the materials; comprehensive pH and temperature studies were performed only for CR, the primary target pollutant.

(2) Effect of Solution pH on CR Adsorption

The pH of CR solutions was adjusted to 4, 6, 8, and 10 using 0.1 mol L^−1^ HCl or NaOH solutions. This process was conducted at 30 °C for 6 h with TAPI-DW.

(3) Effect of Temperature on CR Adsorption

Adsorption of CR (200 mg L^−1^, 50 mL) by TAPI-DW was tested from 30 °C~ 45 °C for 6 h.

(4) Preferential Adsorption

The preferential adsorption capability is crucial for practical applications [21]. TAPI-DW (0.033 g) was added to binary dye mixtures: (i) 50 mg L^−1^ CR + 10 mg L^−1^ MB and (ii) 10 mg L^−1^ MB + 10 mg L^−1^ MO. For comparison, adsorption from a 50 mg L^−1^ CR solution was also performed. Samples were taken at different time intervals, filtered, and analyzed by UV–vis spectroscopy over the 300–800 nm range to monitor preferential adsorption.

(5) Effect of Coexisting Ions

The influence of ionic strength was investigated by adding different salts (KCl, NaCl, CaCl_2_, NaHCO_3_, Na_2_CO_3_, Na_2_SO_4_) at concentrations of 0, 0.01, and 0.02 mol L^−1^ to CR solutions (200 mg L^−1^). TAPI-DW (~0.033 g) was added to each solution. A CR solution without added salts served as the blank control.

(6) Effect of Humic Acid (HA)

CR solutions (200 mg L^−1^) containing HA at concentrations of 0, 5, 10, 15, and 20 mg L^−1^ were prepared. Adsorption was conducted with TAPI-DW at 30 °C for 6 h. The blank control was a CR solution without HA.

## Figures and Tables

**Table 1:** Fitting parameters of TAPI-DW adsorption kinetic model for CR.

Kinetic Model	Parameters			
Pseudo-first-order	Experimental Q_e_ (mg/g)	Theoretical Q_e_ (mg/g)	K_1_ (1/min)	R^2^
	173.0	157.63	0.0284	0.6342
Pseudo-second-order	Experimental Q_e_ (mg/g)	Theoretical Q_e_ (mg/g)	K_2_ (g/mg·min)	R^2^
	173.0	169.10	2.5583	0.8172

**Table 2:** Fitting parameters of TAPI-DW adsorption isotherm model for CR.

Isotherm Model	Langmuir			Freundlich		
Parameters	q_max_ (mg/g)	K_L_ (L/mg)	R^2^	K_F_ (L/mg)	1/n	R^2^
30 °C	206.83	0.1362	0.7537	61.0396	0.2462	0.9797

**Table 3:** Comparison of CR adsorption performance of various adsorbents.

Adsorbent	q_max_ (mg/g)	Ref.
TAPI-DW	206.83	This work
WC–CTAB	25.84	[70]
SAL	74.4	[71]
KA/CEL	130.7	[72]
CB-comp	167.25	[73]
WC@PHTA	549.45	[74]
CU/TETA-0.25 aerogel	575.7	[75]
UiO-67-NH_2_@CA	759.21	[76]

## Data Availability

All data and materials are available on request from the corresponding author. The data are not publicly available due to ongoing research using part of the data.
